# Different levels of estradiol are correlated with sexual dysfunction in adult men

**DOI:** 10.1038/s41598-020-69712-6

**Published:** 2020-07-29

**Authors:** Tong Chen, Fei Wu, Xianlong Wang, Gang Ma, Xujun Xuan, Rong Tang, Sentai Ding, Jiaju Lu

**Affiliations:** 1 Department of Urology, Shandong Provincial Hospital Affiliated to Shandong First Medical University, 324 Jingwu Road, Jinan, 250021 Shandong People’s Republic of China; 2 Center for Reproductive Medicine, National Research Center for Assisted Reproductive Technology and Reproductive Genetics, The Key Laboratory for Reproductive Endocrinology of Ministry of Education, Shandong University, Jinan, 250021 Shandong People’s Republic of China; 3 Department of Pediatric Surgery, Shanghai Children’s Hospital, Shanghai Jiao Tong University, Shanghai, 200062 People’s Republic of China; 4 Department of Urology, Shandong Provincial Qianfoshan Hospital, Jinan, 250002 Shandong People’s Republic of China

**Keywords:** Urogenital diseases, Sexual dysfunction

## Abstract

Ejaculatory dysfunction, including premature ejaculation (PE) and delayed ejaculation (DE), as well as erectile dysfunction (ED), constitute the majority of male sexual dysfunction. Despite a fair amount of data on the role of hormones and erection and ejaculation, it is inconclusive due to controversy in the current literature. To explore the correlation of male sexual dysfunction with hormonal profile, 1,076 men between the ages of 19–60 years (mean: 32.12 years) were included in this retrospective case–control study; 507 were categorized as ED, PE and DE groups. Five hundred and sixty-nine men without sexual dysfunction were enrolled in the control group. The background characteristics and clinical features of the four groups were collected and analyzed. The estradiol value was significantly elevated in the ED group than the control group (109.44 ± 47.14 pmol/L vs. 91.88 ± 27.68 pmol/L; *P* < 0.001). Conversely, the DE group had significantly lower level of estradiol than control did (70.76 ± 27.20 pmol/L vs. 91.88 ± 27.68 pmol/L; *P* < 0.001). The PE group had similar level of estradiol (91.73 ± 31.57 pmol/L vs. 91.88 ± 27.68 pmol/L; *P* = 0.960) but significantly higher level of testosterone (17.23 ± 5.72 nmol/L vs. 15.31 ± 4.31 nmol/L; *P* < 0.001) compared with the control group. In conclusion, elevated serum testosterone concentration was an independent risk factor for PE. Besides, there was a progressively increasing graded-distribution of estradiol values from DE to PE and ED groups.

## Introduction

Sexual dysfunction is a common clinical entity worldwide and has a deleterious role in quality of life for the couple^1^. In men, erectile dysfunction (ED) and ejaculatory dysfunction are the most reported sexual dysfunction. According to the timing of ejaculation, ejaculatory dysfunction can be classified into premature ejaculation (PE) and delayed ejaculation (DE)^2^. ED is involved in pathophysiological alterations via neurogenic, psychoneurologic, vasculogenic, endocrine and iatrogenic pathways. In contrast, the etiology of ejaculatory dysfunction is under-reported and the understanding of hormonal control in ejaculatory dysfunction is still in its infancy.

Hormones can regulate many aspects of male reproduction. Endocrine disorders, including hypogonadism, thyroid diseases and hyperprolactinaemia, has been implicated in the pathogenesis of ED^3^. Testosterone is the major hormonal regulator of penile development and physiology, and affects both the central and peripheral levels of the ejaculatory process^1^. Aromatase is responsible for the conversion of testosterone to estrogen and localized abundantly in the male reproductive system. Levels of estradiol have been demonstrated to be correlated with the incidence and severity of ED^4^, which could be explained by two hypotheses. One hypothesis is that the imbalance between estradiol and testosterone decreases the relaxation of cavernosal smooth muscle via an NO-mediated pathway^5^. The other hypothesis is that estrogen could antagonize the effect of testosterone through the sympathetic and parasympathetic nervous system, which in turn influence erectile function^6^. Apart from the effect on erectile function, estrogen also influences ejaculatory function. In mice, the expression pattern of estrogen receptor (ER) is unique throughout male reproductive tract other than the epididymis. In the epididymis, both ERα and ERβ are expressed^7^. In doing so, epididymal contractility, critical for the first step of emission phase during ejaculation, is regulated by estradiol^8^. ERα or aromatase knockout male mice displayed decreased intromissions and ejaculations compared with wild-type controls^9,10^. In contrast to these findings, several case reports indicated that sexual behavior did not change in men lacking ERα or aromatase^11,12^. Therefore, the influence of ERα or aromatase in males remains controversial.

Collectively, despite a fair amount of data on the role of hormones and erection and ejaculation, it is inconclusive due to controversy in the current literature. Besides, no prior study has investigated the relationship between estradiol and ejaculatory dysfunction in men to date. We hypothesized that testosterone and estradiol might be involved in the regulation of erection and ejaculation in men. To investigate the correlation of hormonal profile with ED, PE, and DE, we conducted a retrospective case–control study with 1,089 men at our institution.

## Materials and methods

### Study population

Between May 2016 and April 2018, adult men with ED, PE or DE who firstly attended andrology clinic of the hospital were enrolled in the current study. Exclusion criteria were: untreated endocrine disorders, psychiatric disorder, anatomical penile abnormality, alcohol or drug abuse in the previous two years, and taking drugs (pseudoephedrine, antidopaminergics, testosterone preparations, serotonin reuptake inhibitors, and antihypo/hyperthyroidism drugs) which might influence intra-vaginal ejaculation latency time (IELT) or hormonal values. Participants were dissuaded from drinking alcohol before sexual intercourse. The coincidence of ED and PE is common in clinical practice, and patients with ED and PE were not taken into the present study. During the same period, subjects from the control group were randomly selected among participants without sexual dysfunction who carried out a health examination before undergoing in-vitro fertilization (IVF). Clinical features of the participants, including background information and hormonal profile, were collected. The study protocol was approved by the ethical committee at Center for Reproductive Medicine, Shandong University. Informed consent was obtained from all participants for this study. Besides, all methods were carried out in accordance with relevant guidelines and regulations.

### Assessment of ED, PE and DE

All participants possessed a stable, monogamous, heterosexual relationship with the partner, and the disease lasted for at least six months. The female partners of the subjects were proposed to apply a calibrated stopwatch to measure IELT. ED is the persistent inability to obtain or maintain an adequate erection to enable satisfactory sexual performance. A patient was diagnosed with ED when IIEF-5 score was smaller than 22^13^. During the screening period, the diagnosis of PE was confirmed when IELT results of sexual intercourse suggested a baseline IELT prior to or within one minute of vaginal penetration at least three times^14^. Furthermore, as the median IELT was 5.4 min in healthy men^2^, the diagnosis of DE was confirmed when the baseline IELT was longer than 25 min (mean plus two standard deviations) and the patient ceased sexual activity due to irritation, exhaustion, partner request or erection loss^15^.

### Measurement of hormonal parameters

For the determination of the hormonal profile, participants with overnight fast were arranged in sitting posture for 30 min before sampling, and then blood samples were drawn from the antecubital veins between 8 and 10 am. Serum samples were analyzed for follicle-stimulating hormone (FSH), luteinizing hormone (LH), prolactin (PRL), total testosterone (T), estradiol (E_2_), and thyroid-stimulating hormone (TSH). When abnormal values appeared, a second sample of the blood was obtained for the re-assessment. The hormonal levels were investigated using the Cobas 6,000 analyzer series (Roche Diagnostics GmbH, Mannheim, Germany) in the laboratory department of the hospital. Among these hormones detected, serum estradiol levels were measured using Elecsys estradiol III kit (Roche Diagnostics Ltd., Shanghai, China). The lower and upper detection limits for estradiol were 28.0 and 156.0 pmol/L, respectively. In addition, the intra-assay coefficient of variation was below 10%, and inter-assay coefficient of variation was below 15%.

### Statistical analysis

We ran the data analysis using SPSS (version 22.0, Chicago, IL, USA) and two-sided *P* values < 0.05 were considered statistically significant. Data were assessed for normality using the Kolmogorov–Smirnov test. The population of the current study was categorized as four groups, namely, ED, PE, DE and control groups. The results were reported as mean ± standard deviation (SD) for continuous variables and number with percentage for categorical variables. One-way ANOVA followed by Student–Newman–Keuls post-hoc test was used to assess the continuous variables. Kruskal–Wallis method was applied for the analysis of skewed variables. The categorical variables were analyzed using Pearson χ^2^ test. For the comparison of differences between two groups, means of normally distributed parameters were analyzed using Student’s *t* test. The contribution of different variables to ED, PE and DE groups was determined using multivariable logistic regression. The odds ratio (OR) was explained as the measurement of correlation.

## Results

### Patient characteristics and hormonal profile

**Figure 1:**
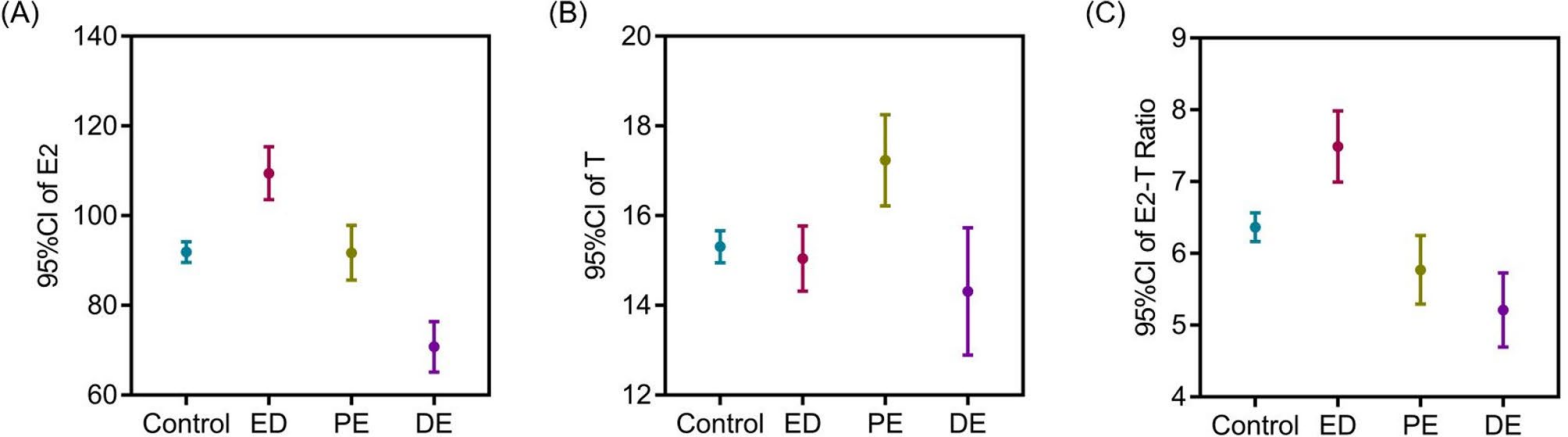
Error bar charts of E_2_, T and E_2_-T ratio among control, ED, PE and DE groups. (**A**) The means and 95% CI of E_2_ among four groups; (**B**) The means and 95% CI of T among four groups; (**C**) The means and 95% CI of E_2_-T ratio among four groups. E_2_-T ratio: estradiol to testosterone ratio. *CI* confidence intervals.

Among 507 men with sexual dysfunction, 277 (53.1%) complained of ED, whereas 124 (23.8%) and 106 (20.3%) declared PE and DE, respectively. Besides, 569 men without sexual dysfunction were recruited in the control group. Table 1 indicates the baseline information of the participants. Figure 1 illustrates the means and 95% confidence interval (CI) of E_2_, T and E_2_-T ratio among control, ED, PE and DE groups, respectively. The comparison of hormonal profile among groups is summarized in Table 2. Testosterone values in the PE group were significantly higher than those in the control group (17.23 ± 5.72 nmol/L vs. 15.31 ± 4.31 nmol/L; *P* < 0.001). Furthermore, estradiol levels were significantly higher for patients with ED (109.44 ± 47.14 pmol/L; *P* < 0.001), but significantly lower for patients with DE (70.76 ± 27.20 pmol/L; *P* < 0.001) compared with those in the control group (91.88 ± 27.68 pmol/L). The ED group had a significantly higher E_2_-T ratio than control (7.49 ± 3.96 × 10^−3^ vs. 6.37 ± 2.41 × 10^−3^; *P* < 0.001) did. Conversely, the E_2_-T ratio was significantly decreased for patients with PE (5.77 ± 2.46 × 10^−3^; *P* = 0.021) and DE (5.21 ± 2.42 × 10^−3^; *P* < 0.001) than for patients in the control group (6.37 ± 2.41 × 10^−3^).

**Table 1:** Background characteristics of the participants among groups.

	Control (n = 569)	ED (n = 277)	PE (n = 124)	DE (n = 106)	*P*-value*
Age (years) mean ± SD	32.33 ± 5.69	31.73 ± 6.19	31.84 ± 6.13	32.19 ± 6.46	0.559
BMI (kg/m^2^) mean ± SD	26.16 ± 4.35	26.78 ± 4.57	25.83 ± 4.53	26.77 ± 4.42	0.163
Smoking n (%)	111 (19.5)	68 (24.5)	28 (22.6)	26 (24.5)	0.322
Hypertension n (%)	46 (8.1)	34 (12.3)	13 (10.5)	10 (9.4)	0.271
Diabetes n (%)	12 (2.1)	13 (4.7)	4 (3.2)	3 (2.8)	0.251
IELT (sec) mean ± SD	359.23 ± 199.45	339.49 ± 170.81	36.67 ± 11.01^a,b^	2,357.33 ± 521.62^a,b,c^	< 0.001
IIEF-5 (score) mean ± SD	23.69 ± 0.95	10.41 ± 4.86^a^	23.19 ± 1.07^b^	23.13 ± 0.99^b^	< 0.001

Data are shown as mean ± SD for continuous variables and number with frequency for categorial variables.

*There were two kinds of *P*-values, one for the ANOVA test for continuous variables, and another for the chi-square test for categorical variables.

*BMI* body mass index, *IELT* Intra-vaginal Ejaculation Latency Time, *IIEF score* International Index of Erectile function scores, *SD* standard deviation.

^a^There was a significant difference compared with data from the control group.

^b^There was a significant difference compared with data from the ED group.

^c^There was a significant difference compared with data from the PE group.

**Table 2:** Hormonal profile of the participants among groups.

	Control (n = 569)	ED (n = 277)	PE (n = 124)	DE (n = 106)	*P*-value
FSH (IU/L)	5.22 ± 2.47	5.20 ± 3.59	5.34 ± 3.36	5.25 ± 3.42	0.978
LH (IU/L)	4.93 ± 1.72	4.91 ± 2.49	5.02 ± 2.35	4.64 ± 2.25	0.535
T (nmol/L)	15.31 ± 4.31	15.04 ± 6.16	17.23 ± 5.72^a,b^	14.31 ± 7.33^c^	< 0.001
PRL (ng/mL)	11.66 ± 4.45	11.72 ± 5.38	12.18 ± 4.90	11.72 ± 5.63	0.789
E_2_ (pmol/L)	91.88 ± 27.68	109.44 ± 47.14^a^	91.73 ± 31.57^b^	70.76 ± 27.20^a,b,c^	< 0.001
TSH (mIU/L)	2.12 ± 0.91	2.14 ± 1.19	2.17 ± 1.23	2.29 ± 1.12	0.594
E_2_-T Ratio (× 10^−3^)	6.37 ± 2.41	7.49 ± 3.96^a^	5.77 ± 2.46^b^	5.21 ± 2.42^a,b^	< 0.001

Data are shown as means ± SD.

*FSH* follicle-stimulating hormone, *LH* luteinizing hormone, *T* total testosterone, *PRL* prolactin, *E*_2_ estradiol, *TSH* thyroid-stimulating hormone.

^a^There was a significant difference compared with data from the control group;

^b^There was a significant difference compared with data from the ED group;

^c^There was a significant difference compared with data from the PE group.

### Independent risk factors for ED, PE and DE

**Figure 2:**
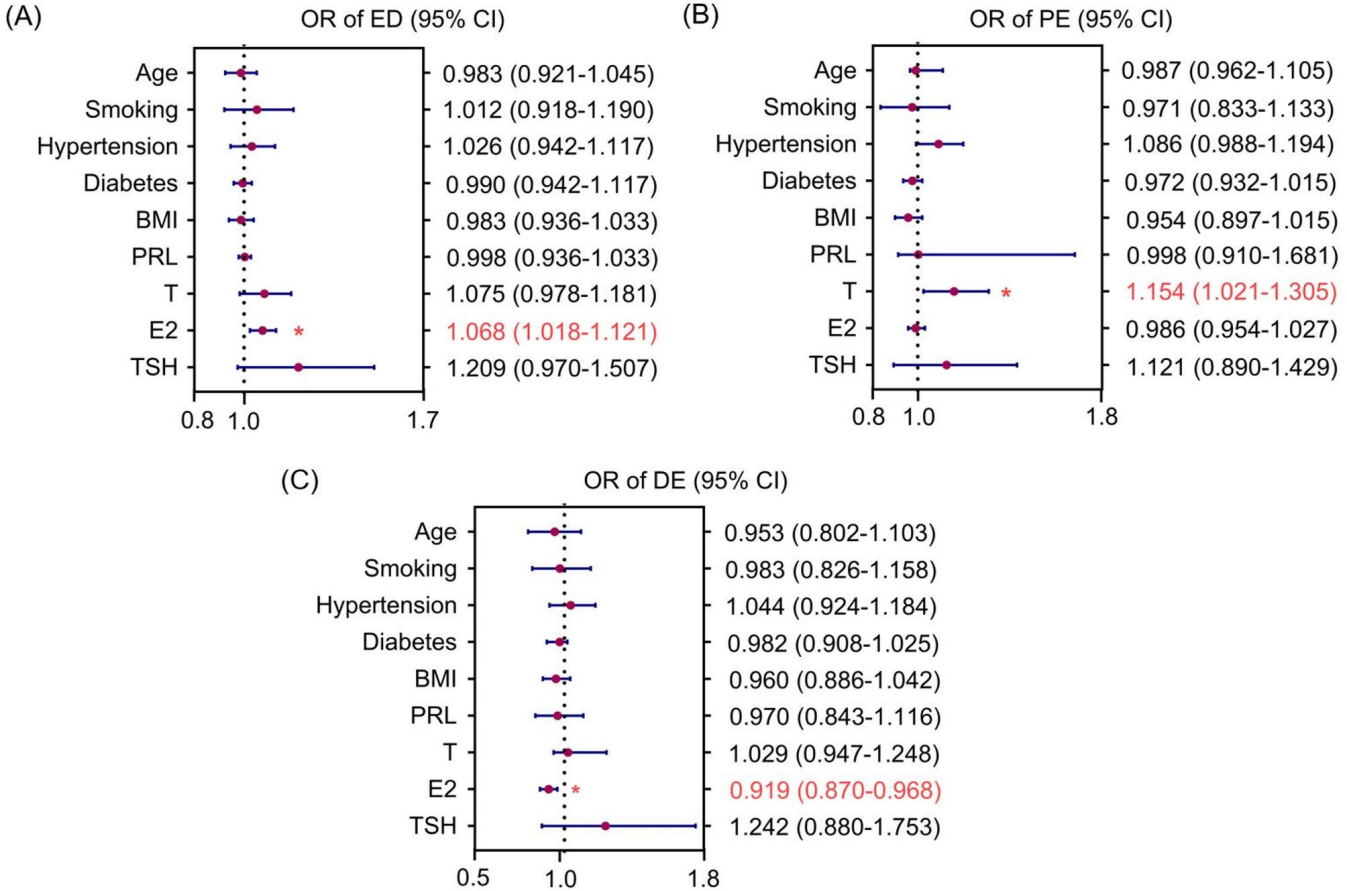
Risk for ED, PE and DE as estimated by different variables. (**A**) The OR of ED for normal controls as references; (**B**) The OR of PE for controls as references; (**C**) The OR of DE for controls as references. *OR* odds ratio; *CI* confidence interval.

Multivariable logistic regression analyses were conducted to explore the ORs for ED, PE or DE. In order to determine independent risk factors, age, BMI, smoking, hypertension, diabetes, testosterone, estradiol, TSH, and prolactin were considered as confounders in the regression models. Figure 2 shows the OR of ED, PE or DE group for controls as the reference. Estradiol was considered as an independent risk factor for ED and DE. Specifically, the OR of ED group versus controls was 1.068 (95% CI 1.018–1.121, *P* < 0.001), and the OR of DE group versus controls was 0.919 (95% CI 0.870–0.968, *P* < 0.001). Furthermore, testosterone was an independent risk factor for PE, and the OR of PE group versus controls was 1.154 (95% CI 1.021–1.305, *P* < 0.001).

### Distinguishing ED, PE and DE using estradiol levels

**Figure 3:**
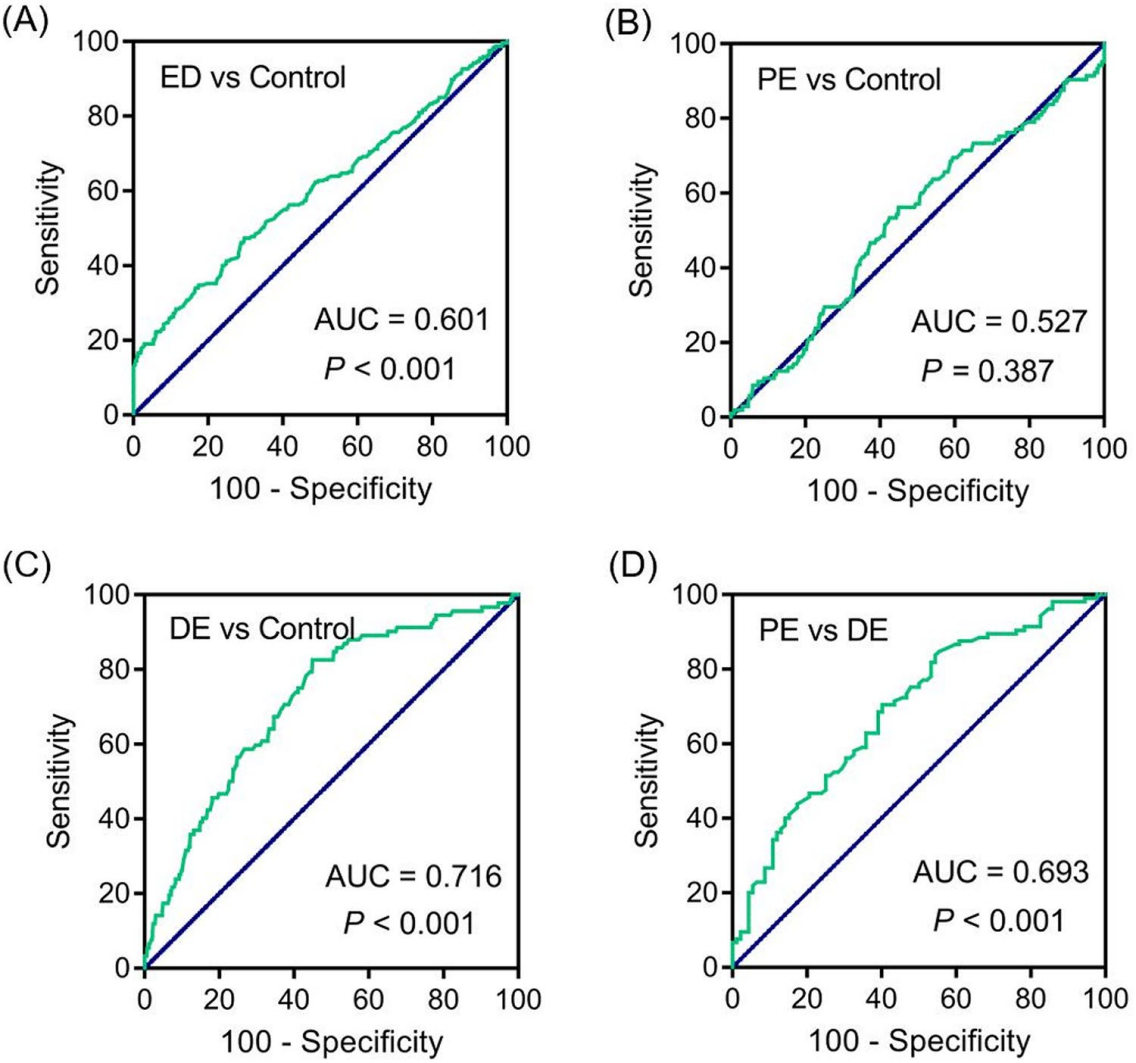
ROC curves of E_2_ with values correlated with AUC. (**A**) The ROC curve of E_2_ to diagnose ED; (**B**) The ROC curve of E_2_ for to diagnose PE; (**C**) The ROC curve of E_2_ to diagnose DE; (**D**) The ROC curve of E_2_ to distinguish between PE and DE.

As the mean level of estradiol was significantly different among groups, the diagnostic effect of the estradiol level for ED, PE, or DE was determined. The receiver operating characteristic (ROC) curve was used to explore area under the ROC curve (AUC) in every group using the estradiol values (Fig. 3). The AUC of ED group is 0.601 and the estradiol level of ED patients is significantly higher than that of men in the control group (Fig. 3A, SE: 0.023, 95% CI: 0.556–0.645, *P* < 0.001). However, estradiol levels were unlikely to distinguish PE patients from normal cases (Fig. 3B, *P* = 0.387). In addition, the AUC of the DE group is 0.716 (Fig. 3C, SE: 0.028, 95% CI: 0.661–0.771, *P* < 0.001). Figure 3D indicates the significantly distinguishing ability of estradiol values between PE and DE groups (AUC = 0.693, SE: 0.037, 95% CI: 0.620–0.766, *P* < 0.001).

**Figure 4:**
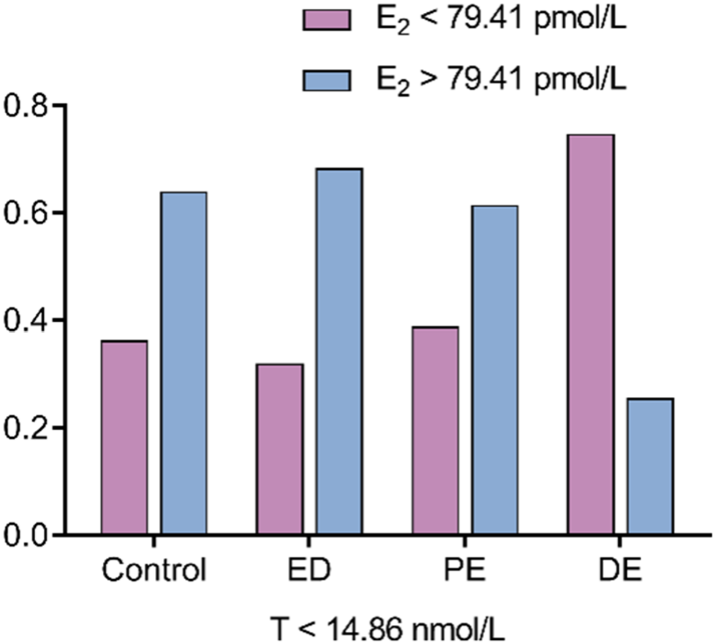
The number of the participants in the four groups. All the participants with T < 14.86 nmol/L were then categorized as two groups based on E_2_ of 79.41 pmol/L.

The best value to distinguish PE from DE was explored using ROC curve and AUC that was 14.86 nmol/L of testosterone (AUC: 0.671, SE: 0.036, 95% CI: 0.600–0.742, *P* < 0.001). Subjects with testosterone > 14.86 nmol/L were more likely to report PE compared with those with testosterone < 14.86 nmol/L (Sensitivity = 0.619, Specificity = 0.726). PE and DE subjects with testosterone < 14.86 nmol/L were further explored using ROC curve followed by AUC of estradiol, and the best value was 79.41 pmol/L (Sensitivity = 0.741, Specificity = 0.613; AUC: 0.703, SE: 0.061, 95% CI: 0.584–0.823, *P* < 0.001). However, levels of estradiol were unlikely to distinguish DE from PE in these patients with testosterone > 14.86 nmol/L (*P* = 0.058). Therefore, we arranged all 528 cases with testosterone < 14.86 nmol/L into eight groups according to the crucial cutoff value of estradiol, and Fig. 4 illustrates the proportion of each group. For cases with testosterone < 14.86 nmol/L, it was likely for DE subjects to possess lower absolute value of estradiol, whereas the other subjects tended to have higher estradiol values.

## Discussion

Overall, our data indicated that significantly higher testosterone values were found in the PE group than in the control group. Levels of estradiol were significantly higher for ED patients, but significantly lower for DE patients when compared with control. The OR of DE was 0.919 for normal controls as the reference. Moreover, the OR of ED was 1.068 for normal controls as references. The best value to distinguish PE from DE was explored using ROC curve and AUC that was 14.86 nmol/L of testosterone.

The pathophysiological role of testosterone in ED has been extensively investigated. However, evidences on the correlation of testosterone levels with ED still remains controversial. Some studies documented that testosterone levels were not associated with ED^16,17^, whereas others reported associations between definitely subnormal levels of testosterone and ED^18^. Our data demonstrated that no statistically significant differences were observed between ED and control groups. Furthermore, as hypothesized, ED was affected by high estradiol levels or the imbalance between estradiol and testosterone^19^. Men with higher estradiol levels had reduced spontaneous erections, decreased nocturnal penile tumescence and greater levels of psychological distresss^20^. In animal models by use of rabbits, increased estrogen levels were associated with ED^21^, and the structure of corpus cavernosum was damaged by administration of exogenous estrogen^19^. The current study, with 88.39% of participants being younger than 40 years old, indicated that elevated estradiol levels were associated with ED in young men. Simultaneously, one interesting finding is that BMIs in the groups did not show a significant difference, but the estradiol level was significantly higher in the ED group. As the majority of aromotase is localized to lipid cells^22^, it is plausible to assume that men with higher estradiol levels are those who are most obese and obesity has clearly been associated with ED^23,24^. To the best of our knowledge, body weight is made up of the sum of fat and lean mass. In men with reduced muscle mass, excess body fat can occur within the normal BMI range. In addition, BMI does not take into account the accumulation of visceral fat that characterizes the most morbigenous form of obesity: central obesity. Waist circumference appears to be more suitable to explain obesity-related health risks than BMI^25^. Also, previous studies demonstrated that waist circumference was superior to BMI in predicting ED^23,24^. Therefore, the reasonable explanation is that the percent of men with central obesity in the ED group might have been higher compared with that in the other groups. Central obesity may not lead to an elevated BMI but can give rise to a higher estradiol level. Similar to our results, recent study reported that elevated estradiol level was significantly associated with ED independent of BMI^26^.

The effect of testosterone on ejaculatory reflex could be explained by central, spinal and peripheral mechanisms^1^. In animal models, elevated testosterone levels decreased serotonin and its metabolite, contributing to PE^18^. Our results reported that PE patients had significantly higher levels of testosterone compared with control. Similar results were reported by other studies^27,28^. Yet, Patrick et al. found no association between PE and testosterone levels^29^. One possible explanation was that saliva samples, instead of blood samples, were applied to measure testosterone levels in Patrick’s study^29^. Although some previous studies reported that low testosterone values were correlated with DE^27,28^, there was no relationship between testosterone values and DE in the present study. This discrepancy could be illustrated by various populations and different eligibility criteria. One advantage of the current study was that the mean age of DE patients and normal controls were 32.19 and 32.33 years, respectively, eliminating the potential influence of aging on testosterone levels. In line with these observations, a recent prospective study reported that normal testosterone levels were found in most of the patients with DE^30^. It has been proposed that thyroid function and prolactin can modulate and interact with the male genital tract even within the normal range^31,32^. Besides, low level of prolactin is associated with a lessened ability to control ejaculation reflex^32^. However, our data demonstrated that there was no significant difference in levels of TSH or prolactin among the groups. Population-related differences might justify this discrepancy, and the association between TSH or prolactin and ejaculatory disorders merits further investigation.

Although high estradiol milieu may adversely affect male sexual function, a moderate estradiol value is beneficial. Men with decreased estradiol levels reported low libido and sexual activity, which could be improved by estrogen administration^33^. Ramasamy et al. demonstrated that in men with decreased libido and testosterone levels below 300 ng/dl, libido was significantly improved when the estradiol level was over 5 ng/dl^34^. ERα, ERβ and aromatase were widely expressed in male genital tract^8^. Particularly, ERα and ERβ were abundantly localized in epididymal cauda, suggesting the role of estrogen in regulating epididymal function^1^. Specifically, in the emission phase during ejaculation, estrogen influences epididymal contractility, thus affecting the latency time^8^. Estradiol could revert hypogonadism-induced downregulation of RhoA/ROCK pathway and restore epididymal contractility^20^. In both rats and humans, ERα, ERβ and aromatase were profusely expressed in male brain, and masculinization of male brain is modulated by locally produced estrogen^20^. Estrogen can influence mood, mental state, cognition, and emotion through an interaction with serotonin receptors^20^. In men with aromatase deficiency, estrogen treatment enhances libido, sexual activity, and erotic fantasies^21^. Animal experiment revealed that aromatase inhibitors significantly reduce ejaculatory activity and sexual motivation, which could be improved by estradiol administration^20^. Decreased intromissions and no ejaculation were observed in ERα or aromatase knockout mice^9,10^. However, a man lacking ERα was reported to have no change in sexual behavior^12^. The current study indicated that estradiol values were significantly decreased for subjects in the DE group than for subjects in the control group. Due to anxiety, fear or attention deficit disorder, DE is characterized by disorders of arousal and sexual cues. Decreased estradiol values might lead to mood disorders before or during sexual intercourse. Also, the contractility of epididymis might be decreased in the setting of low estradiol levels. It was hypothesized that the expulsion phase was mediated by sympathetic excitation, whereas the emission phase was regulated by parasympathetic excitation^6^. In addition, sympathetic excitation might be modulated by estrogen, whereas parasympathetic sexual excitation might be dominated by testosterone^6^. Thus, decreased estradiol values may attenuate the accumulation of sympathetic excitation, which in turn decelerate the expulsion process.

Our study has some limitations. First, estradiol levels in serum samples were not measured using mass spectrometry, which is considered the gold standard method for quantifying estradiol levels^29^. Second, SHBG was not determined, and thyroid function would be better assessed including free triiodothyronine (FT3) and free thyroxine (FT4)^31^. Third, ejaculatory function can be modulated by psychological characteristics to some extent^35,36^, which was not evaluated in this study. Fourth, seminal/infertility status may play a role in male sexual dysfunction^35,37^. However, we did not evaluate seminal/infertility status of the included subjects. Fifth, our data did not include prostate inflammation, which may lead to an altered perception of the ejaculatory reflex and acquired PE^36,38^. Finally, some patients with ejaculatory dysfunction preferred self-reported perceived IELT rather than stopwatch measured IELT. Despite this, it should be noted that self-reported perceived IELT has been proved to be closely correlated with stopwatch measured IELT^39^.

In conclusion, elevated serum testosterone level was an independent risk factor for PE. Moreover, there was a progressively increasing graded-distribution of estradiol values from DE to PE and ED groups.

## References

[1] Corona G, Jannini EA, Vignozzi L, Rastrelli G, Maggi M (2012). The hormonal control of ejaculation. Nat. Rev. Urol..

[2] MD, W. *et al.* A multinational population survey of intravaginal ejaculation latency time. *The journal of sexual medicine* **2**, 492–497 (2005). 10.1111/j.1743-6109.2005.00070.x 16422843

[3] O'connor, D. B. *et al.* The relationships between sex hormones and sexual function in middle-aged and older European men. *J. Clin. Endocrinol. Metab.* **96**, E1577–E1587 (2011). 10.1210/jc.2010-2216 21849522

[4] Schulster M, Bernie AM, Ramasamy R (2016). The role of estradiol in male reproductive function. Asian J. Androl..

[5] El-Sakka AI (2013). Impact of the association between elevated oestradiol and low testosterone levels on erectile dysfunction severity. Asian J. Androl..

[6] Motofei I (2001). The etiology of premature ejaculation starting from a bihormonal model of normal sexual stimulation. Int. J. Impot. Res..

[7] Zhou, Q. *et al.* Localization of androgen and estrogen receptors in adult male mouse reproductive tract. *J. Androl.* **23**, 870–881 (2002). 12399534

[8] Vignozzi L (2008). Regulation of epididymal contractility during semen emission, the first part of the ejaculatory process: a role for estrogen (vol 5, pg 2010, 2008). J. Sex. Med..

[9] Ogawa S, Lubahn DB, Korach KS, Pfaff DW (1997). Behavioral effects of estrogen receptor gene disruption in male mice. Proc. Natl. Acad. Sci. USA.

[10] Bakker J, Honda S, Harada N, Balthazart J (2004). Restoration of male sexual behavior by adult exogenous estrogens in male aromatase knockout mice. Horm. Behav..

[11] Chen Z (2015). Aromatase deficiency in a Chinese adult man caused by novel compound heterozygous CYP19A1 mutations: Effects of estrogen replacement therapy on the bone, lipid, liver and glucose metabolism. Mol. Cell. Endocrinol..

[12] Smith EP (1994). Estrogen resistance caused by a mutation in the estrogen-receptor gene in a man. N. Engl. J. Med..

[13] Rosen RC (1997). The international index of erectile function (IIEF): a multidimensional scale for assessment of erectile dysfunction. Urology.

[14] Se, A. *et al.* An update of the International Society of Sexual Medicine's guidelines for the diagnosis and treatment of premature ejaculation (PE). *J. Sex. Med.* **11**, 1392–1422 (2014). 10.1111/jsm.12504 24848686

[15] McMahon CG, Jannini E, Waldinger M, Rowland D (2013). Standard operating procedures in the disorders of orgasm and ejaculation. J. Sex. Med..

[16] Rhoden EL, Teloken C, Sogari PR, Souto CAV (2002). The relationship of serum testosterone to erectile function in normal aging men. J. Urol..

[17] Kupelian V (2006). Is there a relationship between sex hormones and erectile dysfunction? Results from the Massachusetts Male Aging Study. J. Urol..

[18] Rastrelli G, Corona G, Maggi M (2018). Testosterone and sexual function in men. Maturitas.

[19] Yafi FA (2016). Erectile dysfunction. Nat. Rev. Dis. Primers.

[20] Cooke PS, Nanjappa MK, Ko C, Prins GS, Hess RA (2017). Estrogens in male physiology. Physiol. Rev..

[21] Vignozzi L (2014). Estrogen mediates metabolic syndrome-induced erectile dysfunction: a study in the rabbit. J. Sex. Med..

[22] Longcope C, Kato T, Horton R (1969). Conversion of blood androgens to estrogens in normal adult men and women. J. Clin. Invest..

[23] TS H (2011). Impaired quality of life and sexual function in overweight and obese men: the European Male Ageing Study. Eur. J. Endocrinol..

[24] Janiszewski PM, Janssen I, Ross R (2009). Abdominal obesity and physical inactivity are associated with erectile dysfunction independent of body mass index. J. Sex. Med..

[25] Sa T, Ac H (2011). Waist circumference predicts increased cardiometabolic risk in normal weight adolescent males. Int. J. Pediatric Obesity.

[26] Zuniga KB, Margolin EJ, De Fazio A, Ackerman A, Stahl PJ (2019). The association between elevated serum oestradiol levels and clinically significant erectile dysfunction in men presenting for andrological evaluation. Andrologia.

[27] Corona G (2011). Premature and delayed ejaculation: two ends of a single continuum influenced by hormonal milieu. Int. J. Androl..

[28] Corona G (2010). Different testosterone levels are associated with ejaculatory dysfunction. J. Sex. Med..

[29] Jern P (2014). Associations between salivary testosterone levels, androgen-related genetic polymorphisms, and self-estimated ejaculation latency time. Sex. Med..

[30] Paduch DA (2015). Clinical and demographic correlates of ejaculatory dysfunctions other than premature ejaculation: a prospective, observational study. J. Sex. Med..

[31] Lotti F (2016). Is thyroid hormones evaluation of clinical value in the work-up of males of infertile couples?. Hum. Reprod..

[32] Lotti F (2013). Clinical implications of measuring prolactin levels in males of infertile couples. Andrology.

[33] Finkelstein JS (2013). Gonadal steroids and body composition, strength, and sexual function in men. N. Engl. J. Med..

[34] Ramasamy, R., Kovac, J. R., Scovell, J. M. & Lipshultz, L. I. Words of wisdom. Re: In older men an optimal plasma testosterone is associated with reduced all-cause mortality and higher dihydrotestosterone with reduced ischemic heart disease mortality, while estradiol levels do not predict mortality. *Eur. Urol.* **65**, 844 (2014). 10.1016/j.eururo.2013.12.030 24559901

[35] Lotti F, Maggi M (2018). Sexual dysfunction and male infertility. Nat. Rev. Urol..

[36] Lotti F (2012). Clinical correlates of erectile dysfunction and premature ejaculation in men with couple infertility. J. Sex. Med..

[37] Lotti F (2016). Semen quality impairment is associated with sexual dysfunction according to its severity. Hum. Reprod..

[38] Lotti F (2014). Seminal, clinical and colour-Doppler ultrasound correlations of prostatitis-like symptoms in males of infertile couples. Andrology.

[39] Rosen RC (2007). Correlates to the clinical diagnosis of premature ejaculation: results from a large observational study of men and their partners. J. Urol..

